# Development of a risk score model for the prediction of patients needing percutaneous coronary intervention

**DOI:** 10.1002/jcla.24849

**Published:** 2023-02-17

**Authors:** Xi Wang, Yuping Lin, Feng Wang

**Affiliations:** ^1^ Department of Laboratory Medicine The Affiliated Lihuili Hospital, Ningbo University Ningbo China; ^2^ Department of Cardiovascular Medicine The Affiliated Lihuili Hospital, Ningbo University Ningbo China

**Keywords:** nomogram, percutaneous coronary intervention, predictive model, risk factors

## Abstract

**Background:**

The incidence of coronary heart disease (CHD) is increasing worldwide. The need for percutaneous coronary intervention (PCI) is determined by coronary angiography (CAG). As coronary angiography is an invasive and risky test for patients, it will be of great help to develop a predicting model for the assessment of the probability of PCI in patients with CHD using the test indexes and clinical characteristics.

**Methods:**

A total of 454 patients with CHD were admitted to the cardiovascular medicine department of a hospital from January 2016 to December 2021, including 286 patients who underwent CAG and were treated with PCI, and 168 patients who only underwent CAG to confirm the diagnosis of CHD were set as the control group. Clinical data and laboratory indexes were collected. According to the clinical symptoms and the examination signs, the patients in the PCI therapy group were further split into three subgroups: chronic coronary syndrome (CCS), unstable angina pectoris (UAP), and acute myocardial infarction (AMI). The significant indicators were extracted by comparing the differences among the groups. A nomogram was drawn based on the logistic regression model, and predicted probabilities were performed using *R* software (version 4.1.3).

**Results:**

Twelve risk factors were selected by regression analysis; the nomogram was successfully constructed to predict the probability of needing PCI in patients with CHD. The calibration curve shows that the predicted probability is in good agreement with the actual probability (*C‐index* = 0.84, 95% *CI* = 0.79–0.89). According to the results of the fitted model, the ROC curve was plotted, and the area under the curve was 0.801. Among the three subgroups of the treatment group, 17 indexes were statistically different, and the results of the univariable and multivariable logistic regression analysis revealed that cTnI and ALB were the two most important independent impact factors.

**Conclusion:**

cTnI and ALB are independent factors for the classification of CHD. A nomogram with 12 risk factors can be used to predict the probability of requiring PCI in patients with suspected CHD, which provided a favorable and discriminative model for clinical diagnosis and treatment.

## Graphical abstract

This nomogram predicts the probability of needing PCI in patients with coronary heart disease using 12 indicators. To use the nomogram, the patient variables are positioned on the corresponding axes, a line is drawn to obtain a score, and the *p* value corresponding to the summed total score is the predicted probability. (as marked in red in the Image, the total score for this patient is 698, corresponding to a *p*‐value of 0.985). The graphs are marked with “*” for *p* < 0.05, “**” for *p* < 0.01, and “***” for *p* < 0.001, indicating its significant effect on the predicted results.

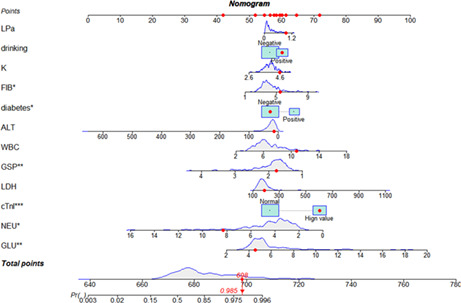

## 1 INTRODUCTION

Coronary heart disease (CHD), one of the leading causes of death worldwide, is defined as atherosclerosis, thromboembolism, or spasm of the blood vessels supplying blood and oxygen to the myocardial cells, causing luminal narrowing or even occlusion, which in turn leads to myocardial ischemia and hypoxia or even necrosis.^1^ The incidence of CHD has been increasing recently and has been getting more common among young individuals. According to the China Cardiovascular Health and Disease Report 2020, cardiovascular disease deaths accounted for the first cause of total deaths among urban and rural residents in China in 2018, with 330 million cardiovascular patients, including about 11.39 million with CHD. The prevalence and mortality of CHD are continuously rising, and seriously threaten human health.^2^ The fast‐paced life, intense work pressure, dietary habits, obesity, and erratic lifestyle are closely related to CHD.^3^, ^4^, ^5^, ^6^

Pharmacologic therapy is fundamental for the stabilization or abatement of coronary atherosclerotic plaques and preventing diseases such as coronary thrombosis, acute myocardial infarction, and sudden cardiac arrest. Revascularization by percutaneous coronary intervention (PCI) or coronary artery bypass grafting (CABG) can further reduce angina, improve life quality, and increase infarct‐free survival.^7^ However, many studies have shown that in most patients with stable angina, the benefits of coronary revascularization are limited to improving the quality of life rather than reducing cardiovascular events.^8^

Coronary angiography is a gold standard for evaluating the degree of coronary artery stenosis in coronary atherosclerotic heart disease. However, it is a risky and invasive procedure for patients. Therefore, in this study, we reviewed the clinical characteristics and laboratory indexes of 454 patients, analyzed the relevant impact factors, and developed a model to predict whether PCI is required, hence exploring a new model to identify CHD requiring PCI economically and feasibly, which will reduce unnecessary medical consumables and alleviate the burden on the healthcare system.

## 2 MATERIALS AND METHODS

### 2.1 Study design and participants selection

From January 2016 to December 2021, a total of 454 patients with CHD were admitted to the cardiovascular medicine department. Of the collected patients, 286 underwent CAG and were treated with PCI, and the remained 168 underwent CAG confirmed the diagnosis of CHD only were put into the control group. According to the clinical symptoms and examination results, the patients in the therapy group were split into three subgroups: 115 patients with chronic coronary syndrome (CCS), 68 patients with unstable angina pectoris (UAP), and 103 patients with acute myocardial infarction (AMI). The 2019 ESC Guidelines for the diagnosis and management of chronic coronary syndromes served as the foundation for the grouping criteria.^9^ The inclusion criteria of current study are CDH diagnosed by CAG, the criteria for intervention with PCI were performed according to the Chinese Guidelines for Percutaneous Coronary Intervention (2016): I. Stable coronary heart disease (SCAD) should be based on the degree of coronary stenosis as a decision basis for whether to intervene, with direct intervention when the lumen stenosis is ≥90%; when the lumen stenosis is <90%, only corresponding intervention if there is evidence of ischemia or a flow reserve fraction (FFR) ≤0.8. II. Patients with non‐ST‐segment elevation acute coronary syndrome (NSTE‐ACS) without ECG ST‐segment elevation are recommended for the first time to have high‐sensitivity troponin testing as their early diagnosis (I, A). III. Emergency coronary angiography should be performed in very high‐risk NSTE‐ ACS patients (<2 h) (I, C); Early CAG is recommended in high‐risk patients, and the choice of an invasive intervention strategy is based on the lesion (<24 h) (I, A).^10^ Exclusion criteria: I. Previous history of coronary stenting or coronary artery bypass surgery; II. Severe heart failure and ventricular arrhythmias; III. Malignancy or history of major surgery. The study protocol was reviewed and approved by the Ethics Committee of Ningbo Medical Center Li Huili Hospital (NO. KY2022PJ188).

### 2.2 Data collection

General clinical data of all patients were recorded, including age, sex, history of hypertension, history of diabetes mellitus, smoking history, and alcohol consumption. After admission, patients underwent echocardiography, and their ejection fraction was recorded. Laboratory tests of routine blood, biochemistry, glycosylated hemoglobin, cardiac markers, and coagulation function were recorded and compiled.

### 2.3 Test method

Routine blood tests were performed using the Sysmex XN‐9000 hematology analyzer. Biochemical indexes were detected using the Siemens ADVIA2400 biochemical analyzer with reagents from Ningbo PREB Biotechnology Company. Glycosylated hemoglobin was detected using the Bio‐Rad D‐100 instrument. High‐sensitivity troponin‐I, creatine kinase, and creatine kinase isoenzyme were detected by Johnson & Johnson VITRO5600 dry biochemical immunoassay machine. The coagulation function was tested using Wolfen ACL‐TOP700 automatic hemagglutination instrument.

### 2.4 Statistical analysis

SPSS 25.0 statistical software was used for data processing and analysis. The measurement data conformed to normal distribution or approximately normal distribution were expressed as *x* ± *s*, and the independent sample t test was used for comparison, Analysis of variance (ANOVA) was used for comparison among the three subgroups, and the chi‐square test was used before ANOVA, and the *F* test was used when the variance was the same, and the Welch test was used when the variance was different; skewed distribution was expressed as *M* (*Q*_*R*_), and rank‐sum test was used for comparison; count data were expressed as the number of cases, and chi‐square test was used for comparison between groups. Univariate and multifactorial analyses were performed to analyze the differences of each index in each group of patients, and then the statistically significant indexes in the analysis results as well as other meaningful indexes were included in the logistic regression analysis, the fitted model was obtained after stepwise regression using R4.1.3 software, and 12 predictors were obtained and plotted in nomogram. The probability of patients with suspected CHD requiring PCI could be obtained by the nomogram scores. *p* < 0.05 was considered a statistically significant difference.

## 3 RESULTS

### 3.1 Comparison of general clinical data between the PCI treatment group and the control group

Male and Diabetic patients account for a higher proportion of PCI cases. There was no statistically significant difference in age, smoking history, drinking history, and hypertension history between the two groups (all *p* values >0.05). As shown in Table 1, the proportion of male and diabetic patients was significantly higher in the PCI treatment group than in the control group (all *p* values <0.05).

**TABLE 1:** Comparison of general clinical data between two groups of patients (*n* = 454).

Variables	Treatment group	Control group	*t*/*χ* ^2^	*p* value
Age (years, *x* ± s)	61.27 ± 11.18	62.85 ± 11.05	1.465	0.935
Gender/(male/female)	207/79	104/64	5.380	0.020
Smoking/(positive/negative)	133/153	64/104	3.046	0.081
Drinking/(positive/negative)	89/197	49/119	0.191	0.662
Hypertension (positive/negative)	182/104	111/57	0.274	0.601
Diabetes/(positive/negative)	76/210	22/146	11.358	0.001

### 3.2 Comparison of testing indexes between two groups of patients

Twenty‐four testing indexes show significant difference between the treated and control groups before coronary angiography. The examination items and laboratory indices from patients without missing indices. The statistically significant differences were found in ejection fraction, creatine kinase, creatine kinase isoenzyme, high‐sensitivity troponin *I*, glucose, glycosylated hemoglobin, fructosamine, alanine aminotransferase, aspartate aminotransferase, lipoprotein a, total bile acids, lactate dehydrogenase, high‐sensitive C‐reactive protein, white blood cell count, absolute neutrophil count, absolute monocyte count, fibrinogen, normal prothrombin time, red blood cell count, ferritin, unconjugated iron, potassium, and creatinine (all *p* values <0.05). For the data high‐sensitivity troponin *I* levels, a skewed distribution and string data types exist, so the data were converted to a categorical variable, that is, 0–0.034 was normal and >0.034 was high, as shown in Table 2.

**TABLE 2:** Test indicators with statistically significant differences between the two groups of patients (*n* = 454).

Variables	Treatment group (286)	Control group (168)	*p* value
EF	0.64 (0.06)	0.66 (0.07)	0.011
CK/(U/L)	89 (82)	82 (48)	0.042
CKMB/(U/L)	14.8 (10.9)	12.65 (6.9)	0.001
hs‐cTnI/(normal/high value)	142/133	117/15	0.000
GLU/(mmol/L)	6.15 ± 2.40	5.18 ± 1.35	0.000
GHbA1/(%)	8.55 ± 1.57	7.9 ± 0.90	0.000
GHbA1c/(%)	6.34 ± 1.36	5.82 ± 0.75	0.000
GSP/(mmol/L)	1.99 ± 0.56	1.85 ± 0.33	0.000
ALT/(U/L)	23 (21)	20 (16)	0.000
AST/(U/L)	23 (19)	20 (7)	0.000
AST/ALT	1.64 ± 1.40	1.11 ± 0.45	0.000
LPa/(g/L)	0.17 (0.25)	0.13 (0.26)	0.029
TBA/(μmol/L)	5.33 ± 4.55	6.98 ± 6.48	0.025
LDH/(U/L)	188 (82)	170 (37)	0.000
hsCRP/(mg/L)	9.52 ± 21.15	3.16 ± 8.25	0.000
WBC/(x10^9^/L)	7.30 ± 2.75	6.29 ± 1.91	0.000
NE#/(x10^9^/L)	4.70 ± 2.48	3.78 ± 1.69	0.000
MO#/(x10^9^/L)	0.55 ± 0.24	0.49 ± 0.17	0.000
FIB/(g/L)	4.29 ± 1.21	3.77 ± 0.78	0.000
NPT/(s)	11.2 ± 0.10	11.31 ± 0.15	0.003
FIB/ALB	0.11 ± 0.04	0.09 ± 0.02	0.000
RBC/(x10^12^/L)	4.54 ± 0.60	4.46 ± 0.49	0.012
FER/(μg/L)	219.5 ± 224	163.4 ± 176.9	0.000
UIBC/(μmol/L)	RBC/(x10^12^/L)	35.83 ± 9.97	0.012
K/(mmol/L)	3.94 ± 0.41	3.96 ± 0.32	0.029
CREA/(μmol/L)	70.85 (23)	67.5 (22.6)	0.029

### 3.3 Comparison of clinical data of the three subgroups of the treatment group

**FIGURE 1:**
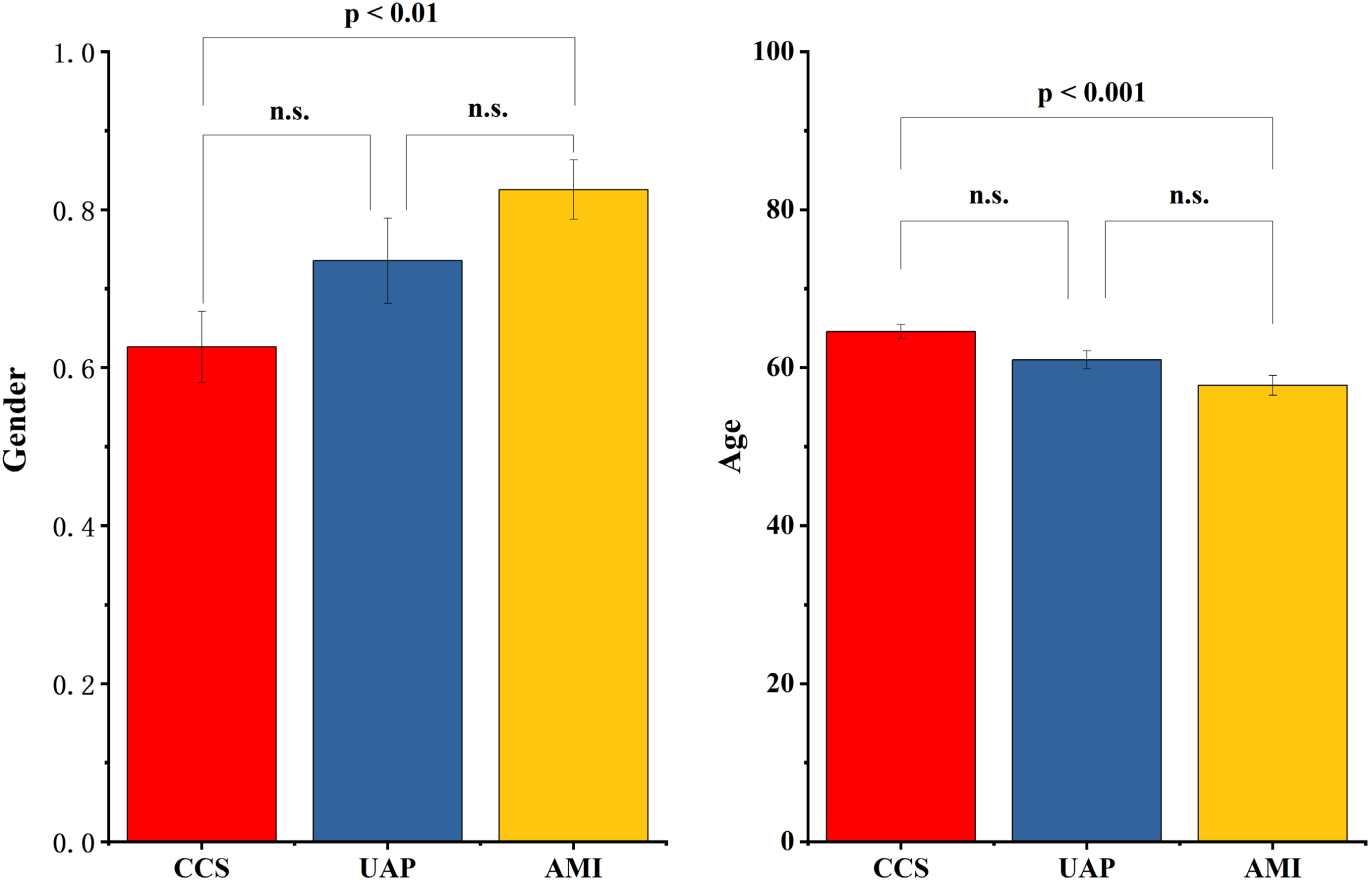
Comparative results of clinical data of patients between the three subgroups of the treatment group.

Among the three subgroups (CCS, UAP, and AMI), gender and age showed statistically significant difference between the CCS and AMI groups (*p* < 0.05), with lower proportion of males in the CCS group and younger patients in the AMI group (Figure 1). This result indicates that older patients are more likely to have CCS and younger patients are more likely to have AMI.

### 3.4 Comparison of laboratory indicators in the three subgroups of the treatment group

**FIGURE 2:**
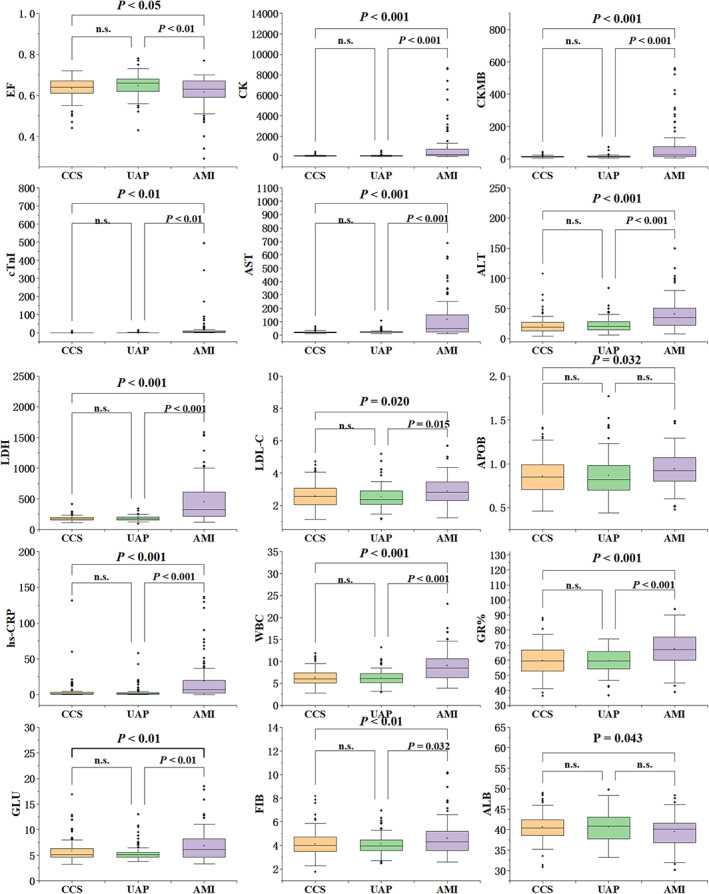
Comparison of test indicators among the three subgroups of patients in the treatment group.

The laboratory indicators such as ejection fraction, creatine kinase, creatine kinase‐MB isoenzyme, troponin I, alanine aminotransferase, aspartate aminotransferase, lactic dehydrogenase, hypersensitive‐C‐reactive protein, leucocyte count, neutrophil count percentage, LDL cholesterol levels, apolipoprotein B, fasting glucose, ferritin, and albumin showed statistically significant among the three subgroups (*p* < 0.05, Figure 2). Ejection fraction and serum albumin were lower in the AMI group than CCS group, and all other test indexes were higher in the AMI group than in CCS and UAP.

### 3.5 Univariate and multifactorial binary logistic regression analysis

**FIGURE 3:**
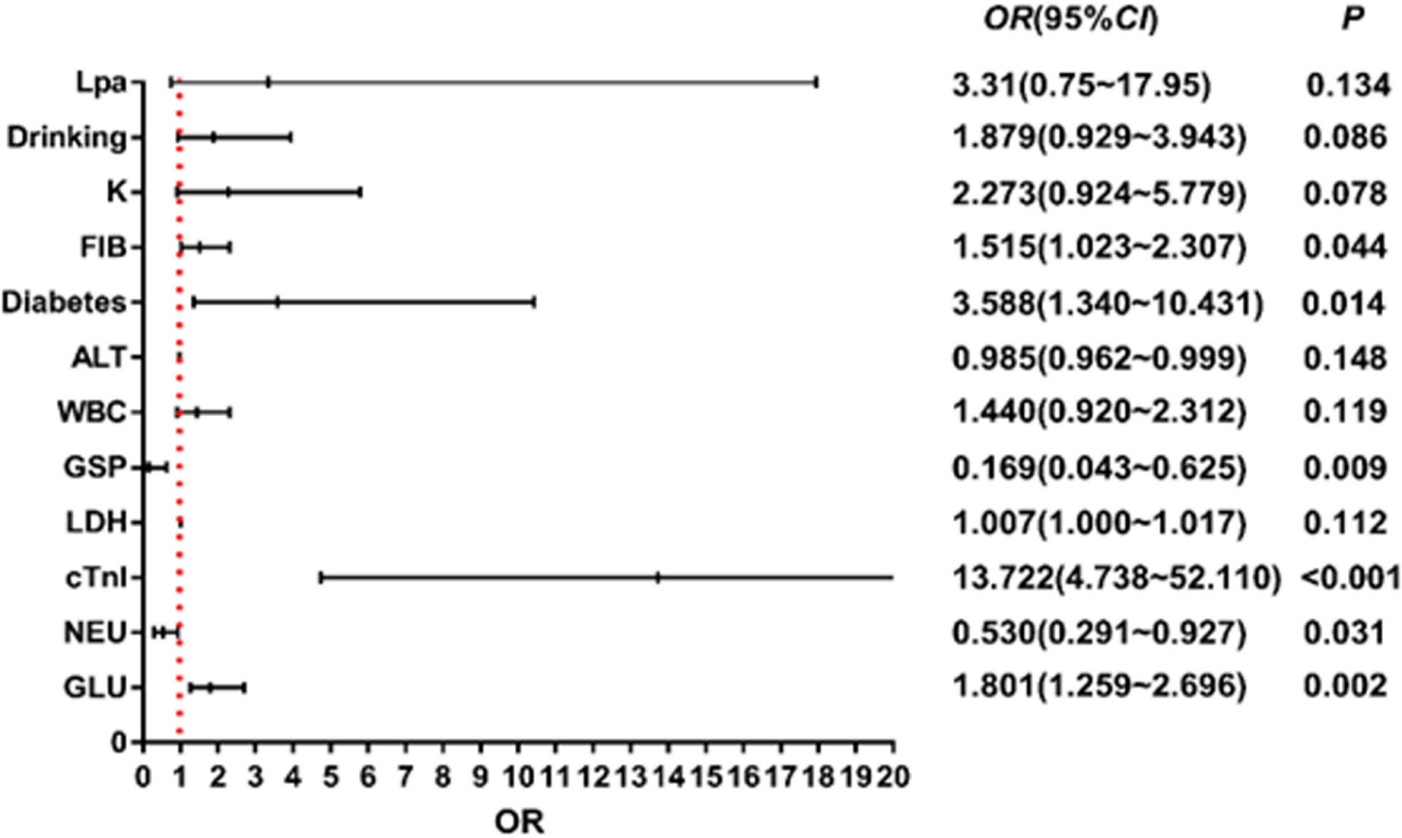
Results of binary logistic regression analysis.

The indicators with significant differences among the aforementioned indicators were used as independent variables. Whether PCI was performed was set as the dependent variable. With univariate logistic regression analysis, previously proved significant indicators showing the severity of CHD but were found insignificant here, such as smoking, alcohol consumption, hypertension, homocysteine, and low‐density lipoprotein, were included in the binary logistic regress. After stepwise regression, 12 predictors were obtained, namely history of diabetes, history of alcohol consumption, potassium, lipoprotein a, fibrinogen, alanine aminotransferase, white blood cell count, fructosamine, lactate dehydrogenase, high‐sensitivity troponin I, absolute neutrophil value, and glucose, and the results of the binary logistic regression analysis were visualized (Figure 3). Among them, the history of diabetes mellitus, high‐sensitivity troponin I, fructosamine, glucose, absolute neutrophil value, and fibrinogen may be independent influencing factors, that is, *p* < 0.05.

### 3.6 Univariate and multifactorial multivariate logistic regression analysis

The indicators with significant differences in the univariate analysis were subjected to univariate logistic regression analysis and multivariate logistic regression analysis, and the results are shown in Table 3. The model fit was good at *p* < 0.05, and the comparison between the CCS and AMI groups showed significance between the two groups in age, cTnI, and ALB.

**TABLE 3:** Results of multivariate logistic regression analysis.

Group		Std. Error	Wald	Sig.	Exp(B)	95% *CI for* Exp(B)	
						Lower bound	Upper bound
CCS	Intercept	4.371	0.365	0.546			
	Age	0.023	4.391	0.036	1.05	1.003	1.098
	EF	3.611	0.062	0.804	2.455	0.002	2908.384
	CK	0.005	1.671	0.196	1.006	0.997	1.015
	CKMB	0.041	1.952	0.162	0.945	0.872	1.023
	cTnI	0.394	4.301	0.038	0.442	0.204	0.956
	ALT	0.019	0.117	0.733	1.007	0.969	1.046
	AST	0.031	1.623	0.203	0.961	0.905	1.021
	LDH	0.006	2.611	0.106	0.991	0.98	1.002
	LDL‐C	0.659	1.007	0.316	0.516	0.142	1.879
	APOB	2.312	0.119	0.73	2.222	0.024	206.243
	hs‐CRP	0.017	0.244	0.621	1.009	0.975	1.044
	WBC	0.124	1.36	0.243	0.866	0.679	1.103
	GR%	0.025	1.122	0.289	0.974	0.929	1.022
	GLU	0.104	0.441	0.506	0.933	0.76	1.145
	FIB	0.248	1.045	0.307	1.288	0.793	2.093
	ALB	0.073	3.987	0.046	1.158	1.003	1.336
	Gender	0.501	0.435	0.509	1.392	0.521	3.72
UAP	Intercept	4.582	0.477	0.49			
	Age	0.023	0.13	0.719	1.008	0.965	1.054
	EF	4.079	2.346	0.126	516.666	0.174	1531533.07
	CK	0.004	0.35	0.554	1.002	0.995	1.009
	CKMB	0.036	0.053	0.817	0.992	0.924	1.065
	cTnI	0.109	1.899	0.168	0.86	0.694	1.066
	ALT	0.019	0.467	0.494	0.987	0.952	1.024
	AST	0.02	0.188	0.665	1.009	0.97	1.049
	LDH	0.006	5.469	0.019	0.987	0.976	0.998
	LDL‐C	0.68	3.587	0.058	0.276	0.073	1.046
	APOB	2.37	1.954	0.162	27.461	0.264	2858.886
	hs‐CRP	0.021	0.304	0.581	0.988	0.948	1.031
	WBC	0.132	1.834	0.176	0.837	0.646	1.083
	GR%	0.026	1.483	0.223	0.969	0.921	1.019
	GLU	0.114	1.119	0.29	0.886	0.709	1.108
	FIB	0.26	2.437	0.119	1.5	0.902	2.494
	ALB	0.073	2.321	0.128	1.118	0.969	1.291
	Gender	0.533	0.009	0.924	0.95	0.335	2.7

*Note*: Reference Category: AMI.

### 3.7 Nomogram drawing and verification

**FIGURE 4:**
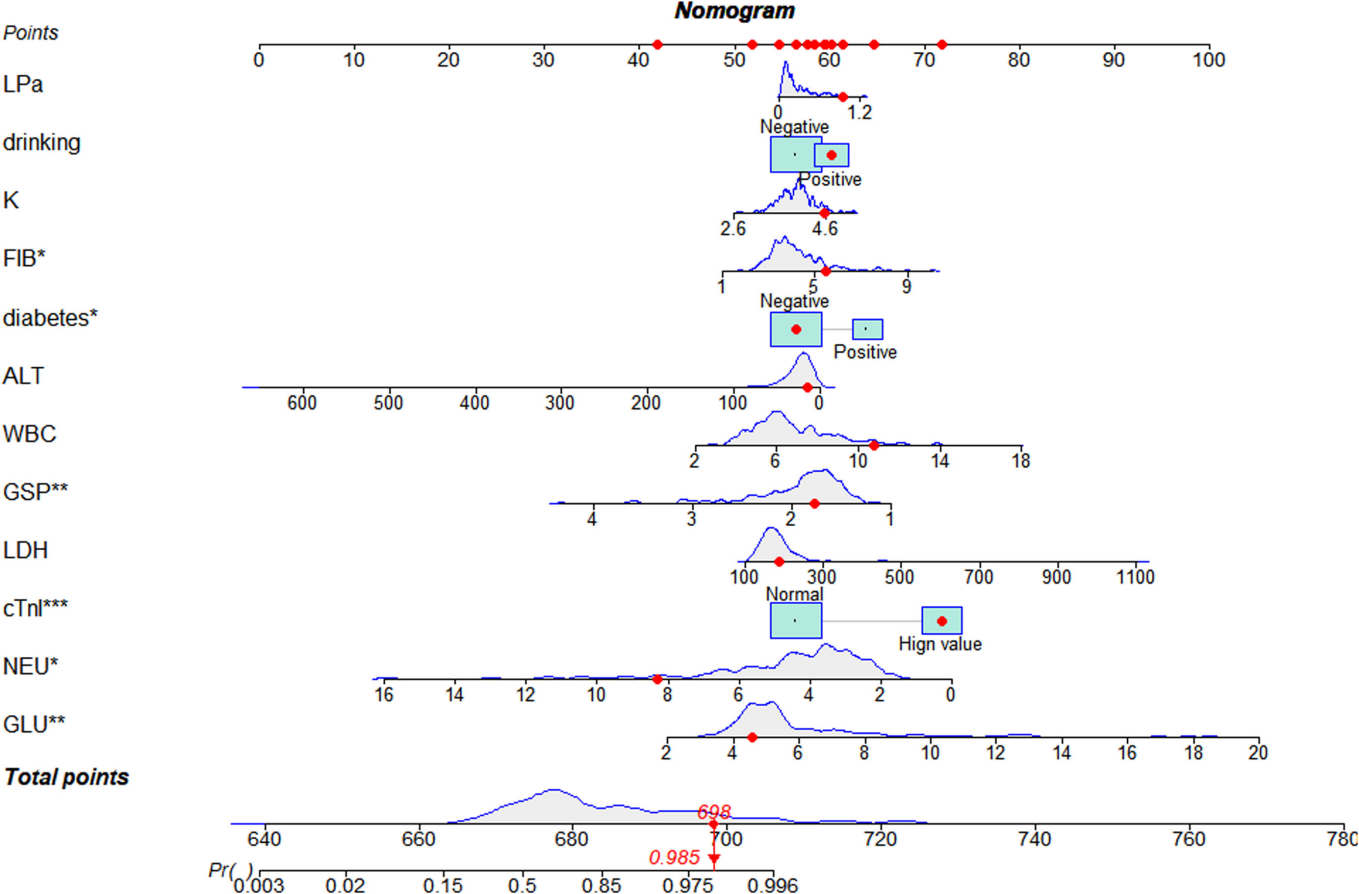
Nomogram for predicting the probability of requiring PCI in patients with coronary heart disease.

**FIGURE 5:**
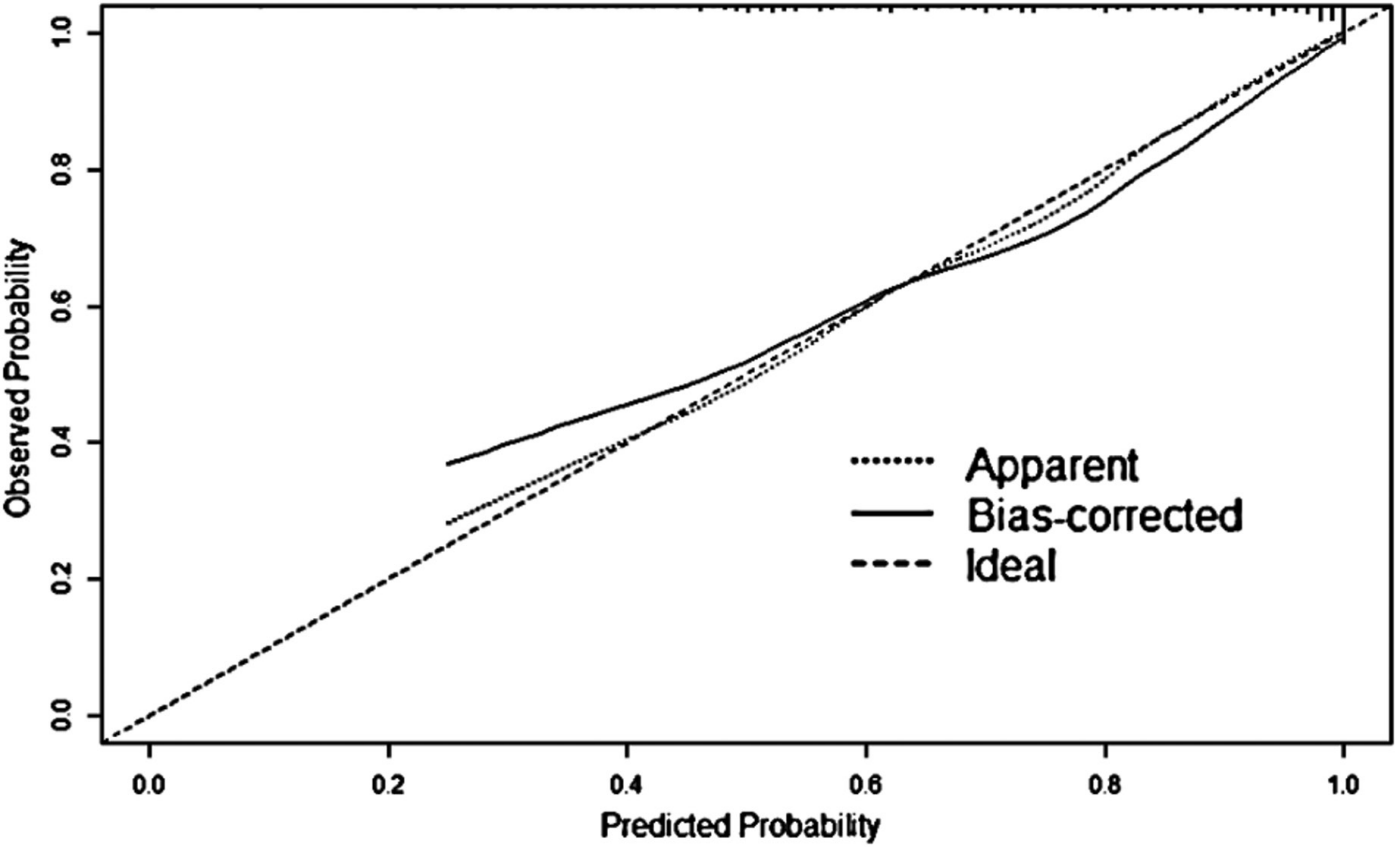
Calibration curve of the prediction model.

**FIGURE 6:**
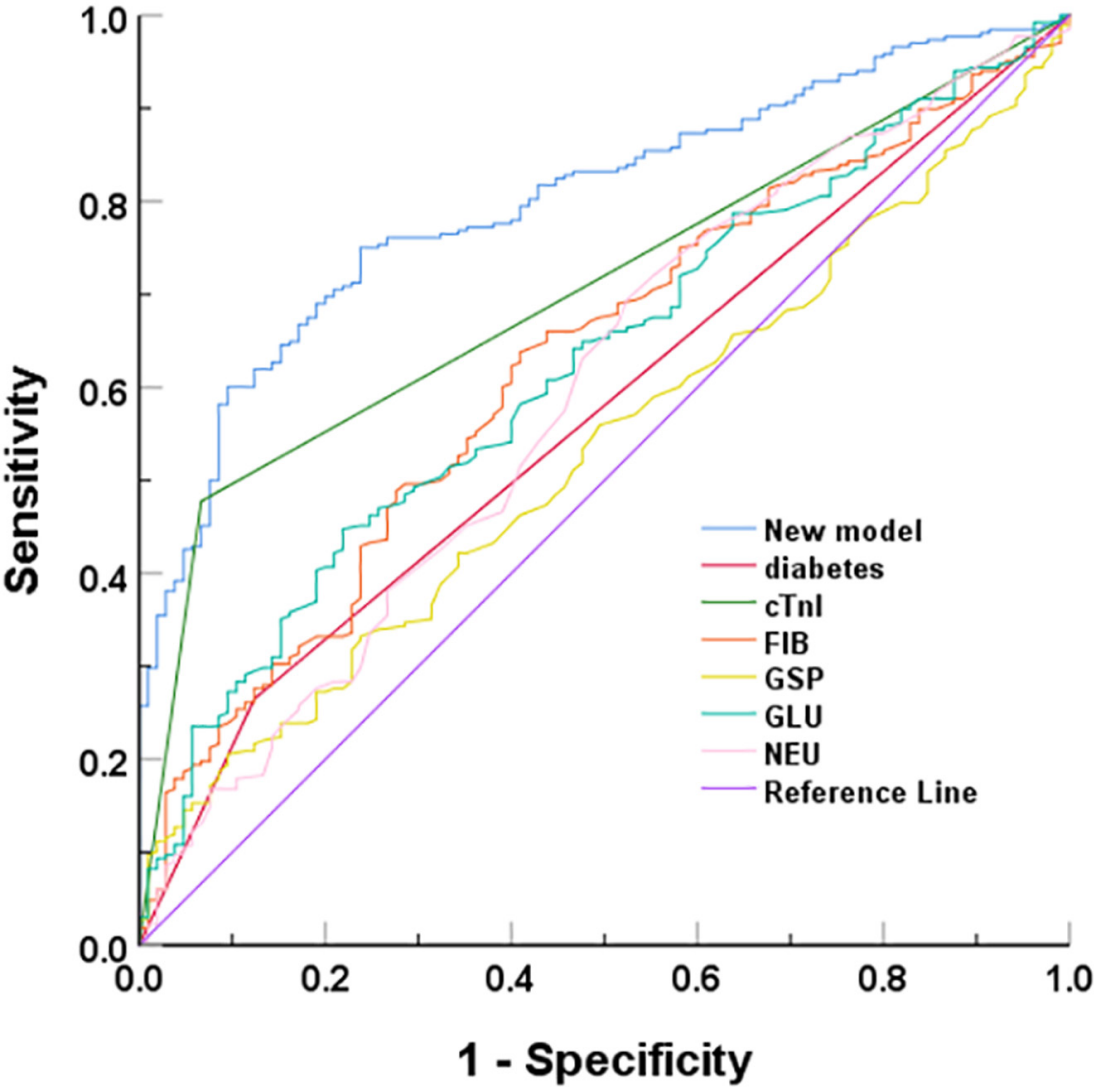
ROC curves of the prediction efficiency of the new model and individual indicators.

Based on the binary logistic regression analysis model, a nomogram was drawn (Figure 4), and the scores of each index could be obtained and summed to obtain the *p*‐value corresponding to the total score, which is the predicted probability of needing PCI in patients with suspected CHD. The calibration curve (Figure 5) was found to be in general agreement with the reference curve, indicating that the predicted probability of occurrence was in good agreement with the actual probability of occurrence. The uncorrected C‐index was 0.84 (95% *CI*: 0.79, 0.89) and the corrected *C‐index* was 0.80, indicating good accuracy of the prediction model (*C‐index* 0.50–0.70 is average accuracy, 0.71–0.90 is good accuracy, and above 0.90 is excellent accuracy), and the results of the goodness‐of‐fit test showed that *p* = 0.372 > 0.05, indicating a good fit. The ROC curve was plotted according to the fitted model results (Figure 6), and the area under the curve was calculated to be 0.801, indicating that the accuracy of the prediction model was good.

## 4 DISCUSSION

Coronary heart disease is a common cardiovascular disease, and the initial diagnosis of CHD can be determined by clinical symptoms, electrocardiogram, and biomarker of myocardial injury. However, CAG is required to confirm the diagnosis of CHD and the degree of coronary stenosis to determine whether the patient needs to undergo PCI. As known, CAG is an invasive procedure, and patients may be allergic to the contrast agent, which will cause severe hypersensitivity reactions and can be life‐threatening.^11^ Many scholars have explored the influencing factors related to the degree of stenosis in patients with CHD, suggesting that the history of diabetes, blood glucose level, white blood cell count, serum troponin, fibrinogen level, fibrinogen to albumin ratio, BNP level, total bilirubin, and uric acid level were independent factors on the severity of coronary artery lesions.^12^, ^13^, ^14^, ^15^, ^16^, ^17^, ^18^ However, to date, there is still a lack of non‐invasive and efficient methods to predict the need for PCI in patients with coronary artery disease. In this study, we reviewed the clinical characteristics and laboratory indices of 454 patients with suspected CHD, analyzed possible influencing factors, and developed predictive models based on the need for PCI. Our results showed that the diabetes history, and levels of troponin *I*, fructosamine, glucose, and absolute neutrophil values were independent PCI markers.

Diabetes is clinically referred to as an equivalent risk for CHD, indicating that diabetes plays an important role in the development of cardiovascular disease.^19^ Numerous studies have confirmed that diabetes mellitus is an important risk factor for CHD. First, elevated blood glucose is a major determinant of arterial stiffness, and chronic hyperglycemia is associated with the accumulation of advanced glycosylation end products (AGEs), which contribute to atherosclerosis and increase the severity of coronary artery lesions. Secondly, oxidative stress exacerbates macrovascular damage in diabetic patients, namely by inducing the production of reactive oxygen species (ROS), which subsequently damages the endothelial system.^11^ Hyperglycemia in diabetic patients also promotes protein kinase C activation and diacylglycerol production, both accelerate the development of atherosclerosis by promoting inflammatory mediators and smooth muscle cell recruitment. It has been shown that the risk of developing CHD in diabetes is highest at any age and is mainly associated with insulin resistance, type 2 diabetes, and metabolic syndrome.^20^ The results of this study also suggest that the history of diabetes and fasting glucose levels, fructosamine are good predictors for PCI in patients with CHD and are the main influencing factors of the severity of coronary artery disease.

Inflammatory responses occur throughout the pathophysiology of the development of coronary atherosclerotic heart disease. Leukocyte counts are positively correlated with the risk of CHD.^13^ Neutrophils are the most abundant circulating leukocytes in the body and play an important role in the inflammatory response. The underlying mechanisms are as follows: (1) neutrophils promote the deleterious effects of TLR2 activation, and this activation leads to persistent local endothelial damage and surface erosion‐associated thrombosis, followed by endothelial cell death or shedding; (2) neutrophil recruitment, thereby expanding, maintaining, and propagating local processes which ultimately lead to endothelial injury and local thrombosis.^21^ The current study suggests that neutrophil count is an important factor in the development process of coronary stenosis and is of great value in the prediction of patients to undergo PCI.

The development of CHD is accompanied by varying degrees of myocardial damage. The measurement of cardiac troponin concentrations has become a central component in the assessment of patients with acute and chronic cardiovascular disease. And studies have shown that the release of troponin is entirely due to irreversible cell death. Troponin can be released into the bloodstream in large amounts by oxidoreductase and protein kinase A phosphorylation, which in turn aggravates the degree of myocardial cell damage, creating a vicious circle and stimulating further progression.^12^ In particular, high‐sensitivity troponin *I* is useful in assessing patients with mild myocardial injury and is a significant predictor for PCI.

Among the three subgroups of the treatment group, ejection fraction, creatine kinase, creatine kinase‐MB isoenzyme, troponin I, alanine aminotransferase, aspartate aminotransferase, lactic dehydrogenase, hypersensitive‐C‐reactive protein, leukocyte count, neutrophil count percentage, LDL cholesterol levels, apolipoprotein B, fasting glucose, ferritin, and albumin basically showed trend changes in the progression of coronary artery disease patients from stable to unstable and then to myocardial infarction. The CCS group and AMI group showed significant differences. Hakan Duman et al.^22^ found that higher CRP levels, lower serum albumin levels, higher CAR, higher neutrophil levels, and troponin I levels were independent predictors of increased thrombotic burden. This study also concluded that lower blood albumin levels and higher cTnI levels were significant predictors of myocardial infarction in individuals.

In conclusion, we found that the history of diabetes mellitus, high‐sensitivity troponin I, fructosamine, glucose, absolute neutrophil value, and *Fibrinogen* were independent factors for predicting PCI. A nomogram using 12 predictors can be used to predict the probability of requiring PCI in patients with suspected CHD, and the accuracy and goodness of fit of our model were verified. Previous studies have shown that hypertension, smoking, and homocysteine are risk factors for CHD, but in this study no statistically significant differences were found between these indicators in patients who underwent PCI or not. We think that these indicators may play an important role in the process of triggering CHD but have little significance for whether to perform PCI; it may also be caused by the relatively small sample size and geographically base of this study, which may generate a possibility of bias, and in the future, we need to enlarge the sample size with multiple centers, and use a machine learning models to improve the prediction efficiency to further improve the predictive ability of PCI for CHD.

## AUTHOR CONTRIBUTIONS

XW, YPL, and FW jointly designed this study and reviewed and revised the article. XW and YPL collected clinical data from CHD patients. XW and FW further collated and preliminarily analyzed the data and conducted statistical analysis and drew the figures and tables of the whole article. XW and YPL wrote the results section of the article, while XW wrote the rest of the article. All authors read and approved the final article.

## FUNDING STATEMENT

No funding.

## CONFLICT OF INTEREST STATEMENT

The authors declare that they have no competing interests.

## CONSENT FOR PUBLICATION

All the participants gave consent for direct quotes from their interviews to be published in this manuscript.

## Data Availability

The data that support the findings of this study are available on request from the corresponding author. The data are not publicly available due to privacy or ethical restrictions.
